# Quantum optical phase, rigged Hilbert spaces and complementarity

**DOI:** 10.1098/rsta.2023.0328

**Published:** 2024-12-24

**Authors:** Khai Bordon, Joan A. Vaccaro

**Affiliations:** ^1^ School of Science, Centre for Quantum Dynamics, Griffith University, Nathan, Australia

**Keywords:** quantum phase operator, Pegg–Barnett phase formalism, rigged hilbert spaces, complementarity

## Abstract

We place Loudon’s quantum treatment of optical phase in *The quantum theory of light* in its historical context, and outline research that it inspired. We show how it led Pegg and Barnett to their quantum phase formalism, explaining the challenges that they overcame to define phase operators and phase eigenstates rigorously. We show how the formalism essentially constructs an extended rigged Hilbert space that supports strong limits of the phase operators and includes their eigenstates. We identify the complementarity structure (consisting of mutually unbiased bases and generators of cyclical permutations) underpinning Pegg and Barnett’s general approach that gives a quantum-classical correspondence free of the ambiguity of Dirac’s commutator–Poisson bracket correspondence.

This article is part of the theme issue ‘The quantum theory of light’.

## 1. Introduction

The book *The quantum theory of light* by Rodney Loudon [1] has been a primary resource in quantum optics for the last 50 years. The first edition appeared in 1973 at a time when the coherence of light had become important experimentally. Indeed, the first maser was built in 1953 followed by the first laser in 1960, and the quantum theory of optical coherence by Glauber and Sudarshan had been developed in the early 1960s [2,3].

However, there were problems with the description of the phase of a single-mode optical field stemming right back to Dirac’s seminal work on quantizing electrodynamics 50 years earlier in 1926 [4]. Their resolution would require not only von Neumann’s casting of quantum mechanics in terms of Hilbert space in 1932 [5], but also the extension known as the *rigged Hilbert space* in the 1950s [6–8]. Unfortunately, a lack of appreciation of the relation between these mathematical developments delayed progress for a further 30 years. It was Loudon’s book in 1973 that played a pivotal role in providing a stepping stone that led to the final resolution in the late 1980s by Pegg and Barnett [9–11] (see also the reviews in [12,13]).

The stimulus provided by Loudon’s book continues to have an ongoing effect. For example, the close relationship between the phase of an oscillator and the time of a clock led Pegg in the 1990s to explore the representation of time in two different contexts: as a relational property in the universe as a whole [14], and as a property, called the *age*, of individual physical systems [15]. Moreover, the lessons learned in developing quantum treatments of phase and time have been further capitalized in a new quantum theory of time that is aimed at exploring the implications of the violation of time reversal symmetry (T violation) by the weak interaction [16,17].

In this paper we place Loudon’s description of optical phase in *The quantum theory of light* in its historical context, and we review important research that it stimulated. Throughout, we find that the way sequences of states are treated played an important part, and led to problems when not tended with sufficient care. We trace, in §2, attempts to treat phase quantum mechanically from the early days of quantum mechanics in the 1920s through to Loudon’s contribution in his book. In §3, we describe Pegg and Barnett’s resolution of the problem in the late 1980s where, notably, sequences of states are addressed appropriately. We also outline infinite dimensional forms of phase operators and states and their relation with other attempts at describing phase. We then describe how the formalism essentially constructs a new rigged Hilbert space that contains the strong limits of the Pegg-Barnett phase eigenstates and supports the strong limit of the phase operators in §4. Next, in §5, we uncover the complementarity structure underpinning Pegg and Barnett’s general approach that gives a quantum-classical correspondence free of the ambiguity of Dirac’s commutator–Poisson bracket correspondence. We close with a discussion and conclusion in §6.

## 2. The historical context

The underpinning of what we now understand as the classical wave theory of light took form in 1865 in Maxwell’s theory of electromagnetism in which light appears to be a propagating electromagnetic wave [18]. In the modern form of Maxwell’s theory, a free electric field 𝐄(𝐫,t) at position 𝐫 and time t satisfying periodic boundary conditions in the Coulomb gauge is given by [1]


(2.1) $$E(r,t)=\sum_{k}\sum_{\lambda }e_{k,\lambda }\;i\omega_{k}[A_{k,\lambda }e^{-i(\omega_{k}t-k.r)}-A_{k,\lambda }^{*}e^{i(\omega_{k}t-k.r)}]$$

where 𝐤 is the wave vector of one of the field’s discrete momentum modes, $\omega_{𝐤}=|𝐤|c$ is the corresponding angular frequency, λ=1,2 indexes a polarization mode, ${𝐞}_{𝐤,\lambda }$ is a unit vector giving the direction of the corresponding polarization mode, and $A_{𝐤,\lambda }$ is the complex amplitude of the corresponding component of the vector potential 𝐀 satisfying 𝐄(𝐫,t)=−∂𝐀/∂t. The associated magnetic field 𝐁 is given by 𝐁=∇×𝐀, however, as the interaction with atoms is dominated by the electric field component of an optical field, we focus solely on the electric field. Expressing the vector potential amplitude in polar coordinates as $A_{k,\lambda }=|A_{k,\lambda }|\;e^{-\;i\phi_{k,\lambda }}$ shows that the electric field ${𝐄}_{𝐤,\lambda }(𝐫,t)$ of each momentum and polarization mode has an angular degree of freedom, $\phi_{𝐤,\lambda }$, called the classical single-mode phase, i.e.


(2.2) $$\begin{array}{lrr} & {𝐄}_{𝐤,\lambda }(𝐫,t)={𝐞}_{𝐤,\lambda }\omega_{𝐤}|A_{𝐤,\lambda }|2\;\sin(\omega_{𝐤}t-𝐤.𝐫+\phi_{𝐤,\lambda }) & \end{array}$$

where $𝐄(𝐫,t)=\sum_{𝐤}\sum_{\lambda }{𝐄}_{𝐤,\lambda }(𝐫,t)$. The phase plays a crucial role in optical interference, coherence and techniques such as homodyne detection.

This approach was challenged by Einstein’s explanation in 1905 of the photoelectric effect as being due to light with angular frequency ω consisting of discrete quantities of energy in multiples of ℏω, reminiscent of Newton’s corpuscular theory. The arrival of quantum mechanics in 1925, built on the work of Planck, Einstein, Bohr, de Broglie, Schrödinger, Heisenberg, Born, Jordan and Dirac, then crystallized the need for a quantum description of electromagnetism. It was provided by Dirac in 1927 who showed that for Einstein’s light quanta (photons) to obey the new Bose–Einstein statistics discovered in 1924 meant that the components $A_{𝐤,\lambda }$ and $A_{𝐤,\lambda }^{*}$ of the vector potential in equation (2.1) need to be replaced with what are now known as annihilation and creation operators ${\hat{a}}_{𝐤,\lambda }$ and ${\hat{a}}_{𝐤,\lambda }^{†}$, respectively, satisfying $[{\hat{a}}_{𝐤,\lambda },{\hat{a}}_{{𝐤}^{\prime },\lambda^{\prime }}^{†}]=\delta_{𝐤,{𝐤}^{\prime }}\delta_{\lambda ,\lambda^{\prime }}$, where $\delta_{i,j}$ is the Kronecker delta. As the classical single-mode phase $-\phi_{𝐤,\lambda }$ is the argument of the complex number $A_{𝐤,\lambda }$, this raises the question of the corresponding quantum phase operator associated with ${\hat{a}}_{𝐤,\lambda }^{†}$.

Interestingly, a year earlier in 1926 Loudon had given what appears to be the earliest attempt at a quantum mechanical treatment of the phase variable of a periodic system [19]. His matrix representations of exponential functions $e^{iw}$ and $e^{-iw}$ of the angle w are not unitary, and he noted that a matrix representation of w itself appeared to be impossible. Despite this, Dirac [4] used the polar decomposition $\hat{a}={\hat{N}}^{1/2}e^{i\hat{\phi }}$ of the annihilation operator $\hat{a}$ of a single field mode in terms of the photon number operator $\hat{N}\equiv {\hat{a}}^{†}\hat{a}$ and a proposed Hermitian phase operator $\hat{\phi }$ where, for convenience, we have dropped the subscripts 𝐤,λ that label the field mode.^1^ He assumed, based on the correspondence between commutators and classical Poisson brackets, $\hat{N}$ and $\hat{\phi }$ to have the simple canonically conjugate relationship


(2.3) $$[\hat{\phi },\hat{N}]=-i\hat{1}$$

where $\hat{𝟙}$ is the identity. Unfortunately, his proposal had clear deficiencies as pointed out by Louisell in 1963 [20], which should not have been unexpected given Loudon’s prior analysis. Indeed, the matrix elements of both sides of equation (2.3) in the photon number eigenstate basis {|n⟩,n=0,1,…} are given by


(2.4) $$(n-n^{\prime })\langle n^{\prime }|\hat{\phi }|n\rangle =-i\delta_{n,n^{\prime }},$$

where $\delta_{n,n^{\prime }}$ is the Kronecker delta, which is inconsistent for $n=n^{\prime }$. The uncertainty relation, $\Delta \hat{N}\Delta \hat{\phi }>1$ [21], that follows from (2.3), where $\Delta \hat{A}=(\langle {\hat{A}}^{2}\rangle -\langle \hat{A}\rangle^{2}{)}^{1/2}$ is the standard deviation in an arbitrary operator $\hat{A}$, is also problematical. In particular, as $\Delta \hat{N}=0$ for a photon number state, the corresponding uncertainty $\Delta \hat{\phi }$ in phase implied by the uncertainty relation is infinite, which complicates the interpretation for an angle variable with a 2π range of values. Louisell suggested that to avoid these issues, the sine and cosine functions of phase might make better choices as phase operators than the phase angle itself, and Susskind and Glogower [22] took up the suggestion a year later. The results are the Hermitian combinations $\hat{\cos}\phi =\frac{1}{2}({\hat{e}}^{i\phi }+{\hat{e}}^{-i\phi })$ and $\hat{\sin}\phi =\frac{1}{2i}({\hat{e}}^{i\phi }-{\hat{e}}^{-i\phi })$ of the operators ${\hat{e}}^{i\phi }$ and ${\hat{e}}^{-i\phi }$ that are defined as being solutions to $\hat{a}={\hat{e}}^{i\phi }{\hat{N}}^{1/2}$ and ${\hat{a}}^{†}={\hat{N}}^{1/2}{\hat{e}}^{-i\phi }$ with the additional conditions $\langle m|{\hat{e}}^{i\phi }|0\rangle =0$ and $\langle 0|{\hat{e}}^{-i\phi }|m\rangle =0$ for number states |m⟩, m=0,1,… that ensure ${\hat{e}}^{i\phi }{\hat{e}}^{-i\phi }=\hat{1}$. Unfortunately, the resulting ${\hat{e}}^{i\phi }$ and ${\hat{e}}^{-i\phi }$ operators don’t commute, are not unitary, and ${\hat{e}}^{-i\phi }{\hat{e}}^{i\phi }=\hat{1}-|0\rangle \langle 0|$. In fact, ${\hat{e}}^{i\phi }$ and ${\hat{e}}^{-i\phi }$ have matrix representations equivalent to those of Loudon’s angle operators $e^{iw}$ and $e^{-iw}$ [19]. Clearly, ${\hat{e}}^{i\phi }$ and ${\hat{e}}^{-i\phi }$ are not exponential functions of a common operator, and so there is no corresponding unique Hermitian phase operator as a result.

A direct repercussion of this situation is the fact that the number states, including the vacuum state, are attributed phase properties that are not consistent with an expected uniform phase distribution. In particular, a classical uniform phase distribution yields sin⁡θ=cos⁡θ=0 and sin2⁡θ=cos2⁡θ=12 where $\bar{g(\theta )}\equiv \int \frac{1}{2\pi }g(\theta )d\theta$ for arbitrary function g(θ), whereas $\langle 0|\hat{\sin\;}\phi |0\rangle =\langle 0|\hat{\cos\;}\phi |0\rangle =0$ and $\langle 0|(\hat{\sin\;}\phi {)}^{2}|0\rangle =\langle 0|(\hat{\cos\;}\phi {)}^{2}|0\rangle =\frac{1}{4}$, and so the operators $\hat{\cos\;}\phi$ and $\hat{\sin\;}\phi$ attribute the vacuum as having a preference for some phase angles over others. As this is independent of any external reference phase, the preference is in absolute terms, which presents a significant conceptual challenge. Despite this, the Susskind–Glogower operators were employed in a range of quantum optical problems [23,24].

Susskind and Glogower attempted to trace the reason for the difficulties. They considered a potential phase operator ϕ associated with a state |θ⟩ in the form of


(2.5) $$|\theta \rangle =\sum_{n=0}^{\infty }\;e^{in\theta }|n\rangle$$

as its eigenstate with corresponding eigenvalue θ. However, they concluded that, as $\langle \theta |\theta^{\prime }\rangle =\sum_{n=0}^{\infty }\;e^{\;in(\theta^{\prime }-\theta )}$ is not a Dirac delta distribution, the states |θ⟩ for different values of θ are not orthogonal, and so the operator ϕ, if it existed, could not be Hermitian. They attributed the reason to the fact that the sum over n in (2.5) is limited to non-negative values only.

However, the real culprit lay much deeper. To reveal it, we need to recall some elementary mathematical analysis and properties of vector spaces. In fact, there is an implicit sequence of states and an associated limit in (2.5) that is often unappreciated due to its commonplace [25]. The situation is analogous to the meaning attached to the symbol $\sum_{n=0}^{\infty }$ in the expression $\sum_{n=0}^{\infty }t_{n}$ for the sequence $t_{0},t_{1},\ldots$ of numbers, which is that the partial sums $S_{m}=\sum_{n=0}^{m}t_{n}$ for m=0,1,… form a convergent sequence over m, and the value of the expression is, in fact, the limit point, i.e. $\sum_{n=0}^{\infty }t_{n}=\lim_{m\to \infty }S_{m}$. A brief review of convergence is given in appendix A(a). The convergence implicit in (2.5) depends on the norm defined for the vector space in which |θ⟩ belongs. Susskind and Glogower did not specify the vector space they were dealing with, which is common practice when it is not a primary concern. Invariably, it would be assumed to be the Hilbert space that von Neumann had identified as being the appropriate one associated with quantum mechanics way back in 1932. Clearly |θ⟩ is not an element of the Hilbert space 𝐇 spanned by the number states {|n⟩:n=0,1,…} simply because it does not have a finite norm given by $\||\psi \rangle \|\equiv \langle \psi |\psi \rangle^{1/2}$ where $\left\langle \psi |\psi^{\prime }\right\rangle$ is the inner product between arbitrary vectors $|\psi \rangle ,|\psi^{\prime }\rangle \in 𝐇$ that is necessary for membership [5]. Moreover, writing the implied limit in (2.5) explicitly as


(2.6) $$\begin{array}{lcr} & |\theta \rangle =\lim_{m\to \infty }|\theta \rangle_{m} & \end{array}$$

where $|\theta \rangle_{m}\equiv \sum_{n=0}^{m}\;e^{in\theta }|n\rangle$ reveals the sequence of vectors $|\theta \rangle_{0},|\theta \rangle_{1},\ldots$ associated with it. This sequence does not converge strongly in 𝐇, i.e. there does *not* exist a number $N_{\epsilon }$ such that $\||\theta \rangle_{m}-|\theta \rangle_{r}\|=|m-r{|}^{1/2}<\epsilon$ for any ϵ>0 for all $m,r>N_{\epsilon }$ and so the limit in (2.6) (and implicit in (2.5)) does not exist. We will revisit this result in §3(b) when we consider a weaker form of convergence.

Although the state |θ⟩ has a divergent norm, Susskind and Glogower did include normalization in their calculation of expectation values in a particular way. As demonstrated by their (27)–(29), they extended the conventional definition $\left\langle \psi |\hat{Q}|\psi \rangle /\langle \psi |\psi \right\rangle$ of the expectation value of an arbitrary operator $\hat{Q}$ for an unnormalized state |ψ⟩∈𝐇 to apply to calculations involving |θ⟩∉𝐇 by implicitly using terms in the sequence of vectors $|\theta \rangle_{0},|\theta \rangle_{1},\ldots$ and taking the limit m→∞ of the result, i.e.


(2.7) $$\begin{array}{lcr} & \langle \hat{Q}\rangle =\lim_{m\to \infty }\;\frac{{,}_{m}\langle \theta |\hat{Q}|\theta \rangle_{m}}{{,}_{m}\langle \theta |\theta \rangle_{m}}. & \end{array}$$

The use of vectors with divergent norms that are excluded from 𝐇, such as |θ⟩, was tolerated by many authors because it was seen to be a necessary part of a complete theory (see e.g. Messiah’s *Quantum mechanics* V1 Ch. V Part III [26]).

The justification for this tolerance could be argued to have stemmed from Dirac’s use of his eponymous δ-function much earlier (although Dirac acknowledged that the δ-function was, at best, an improper function [27], and von Neumann considered it to be a convenient fiction [5]). A mathematical framework to support it had begun construction in the late 1930s [28] initially as the theory of generalized functions [6,29] and later as distribution theory. Dirac’s improper δ-function is nowadays regarded as a distribution. The focus shifted during the 1950s to the nature of the vector spaces that house the functions and their generalizations. This led to the structure that is called the *rigged Hilbert space* [7,8] which embellishes 𝐇 with two new spaces related to it by


(2.8) $$\begin{array}{lrr} & 𝚽\subset 𝐇\subset {𝚽}^{\prime } & \end{array}$$

where 𝚽 is a vector space of well-behaved (e.g. ‘smooth’) test functions and ${𝚽}^{\prime }$ is the vector space of distributions. A brief review of the rigged Hilbert space is given in appendix A. It is no longer necessary to appeal to one’s mathematical *tolerance* to make use of certain vectors of divergent norm in quantum mechanics because they are now recognized as legitimate elements of ${𝚽}^{\prime }$. Indeed, the position and momentum eigenstates are represented in ${𝚽}^{\prime }$. Although distribution theory gradually became better understood in the physics community, the rigged Hilbert space would unfortunately remain largely unappreciated for some considerable time.

This was the situation at the time the first edition of Loudon’s *The quantum theory of light* appeared in print in 1973. Chapter 6 discusses the phase properties of a single-mode field using the Susskind–Glogower operators. Taking a step further, Loudon replaced Susskind and Glogowers’ phase state |θ⟩ in (2.5) with the following revised version [1]


(2.9) $$|\phi \rangle =\lim_{s\to \infty }(s+1{)}^{-1/2}\sum_{n=0}^{s}e^{in\theta }|n\rangle .$$

Although the individual vectors, $|\phi \rangle_{s}\equiv (s+1{)}^{-1/2}\sum_{n=0}^{s}\;e^{in\theta }|n\rangle$, over s are each normalized and despite the fact that $|\phi \rangle_{s}\in 𝐇$ for all s, nevertheless the sequence $|\phi \rangle_{s}$ for s=0,1,… does not converge strongly on 𝐇. Namely, $\||\phi \rangle_{s}-|\phi \rangle_{r}{\|}^{2}=2(1-\sqrt{\left(r+1)/(s+1\right)})$ for s>r and convergence requires that there exists a number $N_{\epsilon }$ such that $\||\phi \rangle_{s}-|\phi \rangle_{r}\|<\epsilon$ for any ϵ>0 for *all* $r,s>N_{\epsilon }$. But, for *any* $N_{\epsilon }>1$, if it existed, we could choose $r+1=2N_{\epsilon }$ and $s+1>3N_{\epsilon }$ (satisfying $r,s>N_{\epsilon }$) which gives (r+1)/(s+1)<2/3 and so $\||\phi \rangle_{s}-|\phi \rangle_{r}{\|}^{2}>2(1-\sqrt{2/3})$, independently of ϵ, proving that required number $N_{\epsilon }$ does not exist and, thus, that the sequence does not converge. This implies that |ϕ⟩∉𝐇. Although the norm of |ϕ⟩ is written as ⟨ϕ|ϕ⟩=1 in Loudon’s (7.29), which implies taking the limit in (2.9) separately of ⟨ϕ| and also of |ϕ⟩, this is presumably not what was intended in general [1]. Indeed, it is evident from his (7.31), (7.32) that the limit is to be applied to *expectation values* rather than individual states $|\phi \rangle_{s}$, as in


(2.10) $$\begin{array}{lcr} & \langle \hat{Q}\rangle =\lim_{s\to \infty }\;{,}_{s}\langle \phi |\hat{Q}|\phi \rangle_{s}, & \end{array}$$

which is consistent with Suskind and Glogower’s method in (2.7). According to the Born probability interpretation, normalization represents the certainty that a vector represents a possible state of a physical system, and as the normalization of $|\phi \rangle_{s}$ for each value of s is specific to the corresponding set $\{|n\rangle {\}}_{s}\equiv \{|n\rangle :n=0,1,\ldots ,s\}$ of number states, the expression ${,}_{s}\langle \phi |\hat{Q}|\phi \rangle_{s}$ in (2.10) unequivocally represents an expectation value for each (s+1)-dimensional subspace of 𝐇 that is spanned by $\{|n\rangle {\}}_{s}$. That is, Loudon is effectively proposing that the value of $\left\langle \hat{Q}\right\rangle$ for a state |ϕ⟩ of well-defined phase ϕ is the limit point of a sequence of expectation values of $\hat{Q}$, each one calculated for the representative state $|\phi \rangle_{s}$ in a *sequence of finite-dimensional subspaces* of 𝐇.

While it is possible to recast (2.7) in the same light, Loudon’s explicit normalization of the phase states in (2.9) draws attention to an underlying sequence of subspaces, and with it a new possibility of an unbounded sequence of finite-dimensional subspace representations of the physical system being considered. Correspondingly, the conceptual chasm between the quantum phase and the rigged Hilbert space narrowed considerably.

However, despite Bohm [7] publishing lecture notes that introduced the rigged Hilbert space framework in an accessible format in 1978, it continued to escape the attention of physicists.^2^ Instead, attempts continued to be made to construct unitary replacements for the Susskind–Glogower operators ${\hat{e}}^{i\phi }$ and ${\hat{e}}^{-i\phi }$ using variations of the conventional Hilbert space. The fact that for an operator to be unitary, it must map every state into another state of the same norm led to proposals for replacing the Susskind–Glogower lowering operator ${\hat{e}}^{i\phi }$ (which lowers all number states, i.e. ${\hat{e}}^{i\phi }|n\rangle =|n-1\rangle$ for n=1,2…, except for the vacuum which it ‘annihilates’, i.e. ${\hat{e}}^{i\phi }|0\rangle =0$) with one that lowers every number state by including states of negative ‘photon’ numbers, and thus negative energy [23,31–33]. While mathematically rigorous, these proposals lack the appeal of being based on a Hilbert space containing only physically meaningful states.

## 3. Pegg and Barnett’s phase formalism

### (a) The formalism

The situation changed in 1989. Pegg and Barnett realized the importance of the finite-dimensional spaces implicit in Loudon’s phase state (2.9) and method of calculating expectation values in (2.10). They cite Bohm’s lecture notes [7] when discussing Loudon’s phase state |ϕ⟩, and so had the advantage of being aware of the implications of the rigged Hilbert space structure [9]. In a nutshell, rather than using a unitary lowering operator that takes the vacuum to a negative photon number state, they instead constructed a *cyclical* version that takes the vacuum |0⟩ up to the highest number state |s⟩ in the space ${𝚿}_{s}$ which they define to be a (s+1)-dimensional subspace of 𝐇 spanned by the set {|n⟩:n=0,1,…,s} of number states [9–11]. The cyclical mapping from one energy eigenstate to the next in either direction, lower or higher, by a unitary operator is represented on a finite-dimensional space without any difficulty. The key to exploiting this property of a finite-dimensional space, however, is developing a satisfactory method to deal with all states that might be encountered in a physically realistic field. Rather than employ the rigged Hilbert space structure directly, Pegg and Barnett chose to use a *limiting procedure* for dealing with the limit s→∞ together with a physically motivated restriction on the state space to what they call *physical states* [9]. This approach is sufficient for deriving any desired quantity representing empirically acquired data, and it allowed them to avoid discussion of mathematical details, nevertheless, we demonstrate below in §4 that it essentially builds the rigged Hilbert space structure in any case.

The Pegg–Barnett formalism [9–11] focuses on Loudon’s phase state in (2.9) for each value of s,


(3.1) $$|\theta \rangle_{s}=(s+1{)}^{-\frac{1}{2}}\sum_{n=0}^{s}e^{in\theta }|n\rangle ,$$

and the corresponding (s+1)-dimensional subspace of 𝐇 spanned by {|n⟩:n=0,1,…,s} that it belongs to, ${𝚿}_{s}$. These states have a natural and simple time development: the initial state $|\theta \rangle_{s}$ evolves to $|\theta -\omega t\rangle_{s}$ after a time t, where ω represents the frequency of the single mode field under consideration. As $|n\rangle =(2\pi {)}^{-1}(s+1{)}^{\frac{1}{2}}\int_{2\pi }\;e^{-in\theta }|\theta \rangle_{s}d\theta$ for n=0,1,…,s, the set of phase states clearly form a basis for ${𝚿}_{s}$, and as there is an uncountable number of different phase states |θ⟩, one for each value of θ in any 2π interval, the basis is over-complete. A subset of (s+1) basis states is constructed as follows. For definiteness, the beginning of an arbitrary 2π interval is labelled $\theta_{0}$ and called the *reference phase*, and a set of (s+1) phase states is defined as^3^


(3.2) $$|\theta_{m}\rangle_{s}=(s+1{)}^{-\frac{1}{2}}\sum_{n=0}^{s}e^{in\theta_{m}}|n\rangle ,$$

where


(3.3) $$\begin{array}{lrr} & \theta_{m}=\theta_{0}+\frac{2m\pi }{s+1}\;\text{for}\;m=0,1,\ldots ,s. & \end{array}$$

The set $\left\{|\theta_{m}\rangle_{s}:m=0,1,\ldots ,s\right\}$ is a complete basis for $\Psi_{s}\;$, as the elements are mutually orthogonal and form a resolution of the identity ${\hat{1}}_{s}\;$, i.e.


(3.4) $${,}_{s}\langle \theta_{m^{\prime }}|\theta_{m}\rangle_{s}=(s+1{)}^{-1}\sum_{n=0}^{s}e^{in(\theta_{m}-\theta_{m^{\prime }})}=\delta_{m,m^{\prime }},\;{\hat{1}}_{s}=\sum_{m=0}^{s}|\theta_{m}\rangle_{\;s}{,}_{s\;}\langle \theta_{m}|.$$

The choice of $\theta_{0}$ determines a particular basis of phase states for the space. The operators representing phase are defined to have the phase basis states as eigenstates. That is, the exponential and Hermitian phase operators are simply [10,11]


(3.5) $$\begin{array}{rl} e^{i{\hat{\phi }}_{\theta }} & =\sum_{m=0}^{s}e^{i\theta_{m}}|\theta_{m}\rangle_{\;s}{,}_{s\;}\langle \theta_{m}|=|0\rangle \langle 1|+|1\rangle \langle 2|+\ldots +|s-1\rangle \langle s|+e^{i(s+1)\theta_{0}}|s\rangle \langle 0| \end{array}$$

(3.6) $$\begin{array}{rl} & =(e^{-i{\hat{\phi }}_{\theta }}{)}^{†}, \end{array}$$

(3.7) $$\begin{array}{lrlr} & {\hat{\phi }}_{\theta } & =\sum_{m=0}^{s}\theta_{m}|\theta_{m}\rangle_{\;s}{,}_{s\;}\langle \theta_{m}|. & \end{array}$$

The exponential phase operators are unitary on $\Psi_{s}\;$, i.e.


(3.8) $$(e^{i{\hat{\phi }}_{\theta }}{)}^{†}e^{i{\hat{\phi }}_{\theta }}=e^{i{\hat{\phi }}_{\theta }}(e^{i{\hat{\phi }}_{\theta }}{)}^{†}=\sum_{n=0}^{s}|n\rangle \langle n|={\hat{1}}_{s}$$

where


(3.9) $$\begin{array}{lrr} & {\hat{𝟙}}_{s}\equiv \sum_{n=0}^{s}|n\rangle \langle n| & \end{array}$$

is the identity operator on ${𝚿}_{s}$. This is due to the *cyclical* ladder properties:^4^


(3.10) $$\begin{array}{rl} e^{i{\hat{\phi }}_{\theta }}|n\rangle & =|n-1\rangle \text{ for }1\leq n\leq s,\;e^{i{\hat{\phi }}_{\theta }}|0\rangle =e^{i(s+1)\theta_{0}}|s\rangle \\ e^{-i{\hat{\phi }}_{\theta }}|n\rangle & =|n+1\rangle \text{ for }0\leq n\leq s-1,\;e^{-i{\hat{\phi }}_{\theta }}|s\rangle =e^{-i(s+1)\theta_{0}}|0\rangle . \end{array}$$

Naturally, the operator ${\hat{\phi }}_{\theta }$ depends on the choice of reference phase $\theta_{0}$ as its eigenvalues range from $\theta_{0}$ to $\theta_{0}+2\pi s/(s+1)$. As shown in (2.4) above, Dirac’s proposal for a Hermitian phase operator suffered from not having a meaningful matrix representation in the number state basis [20]. The Pegg–Barnett Hermitian phase operator ${\hat{\phi }}_{\theta }$ is free from such problems: its diagonal and off-diagonal number state matrix elements are well defined [10].

The procedure for calculating the expectation value of an arbitrary operator $\hat{Q}$ for an arbitrary state |q⟩ entails taking the limit s→∞, where it exists, of the sequence of expectation values $\langle \hat{Q}\rangle_{s}={,}_{s}\langle q|{\hat{Q}}_{s}|q\rangle_{s}$ on ${𝚿}_{s}$ for each value of s where ${\hat{Q}}_{s}$ and $|q\rangle_{s}$ are (s+1)-dimensional representations of $\hat{Q}$ and |q⟩ in ${𝚿}_{s}$. For example, the expectation value of the Hermitian phase operator in (3.7) is given by the limit point $\langle {\hat{\phi }}_{\theta }\rangle =\lim_{s\to \infty }\langle {\hat{\phi }}_{\theta }\rangle_{s}$ of the sequence of values $\langle {\hat{\phi }}_{\theta }\rangle_{0}$, $\langle {\hat{\phi }}_{\theta }\rangle_{1}$, …, $\langle {\hat{\phi }}_{\theta }\rangle_{s}$, …, each of which is calculated for the corresponding space ${𝚿}_{s}$.

The restriction to physical states limits the vectors, $|p\rangle =\sum_{n=0}^{\infty }p_{n}|n\rangle$, to those that can be prepared from the vacuum state by coupling the field mode to a finite energy source for a finite time through a finite interaction coupling. For these states $\langle p|{\hat{N}}^{m}|p\rangle =\sum_{n=0}^{\infty }|p_{n}|n^{m}<\infty$ for any given positive integer value m, i.e. all moments of the photon number are finite [9,37]. This reduces the calculation of expectation values to those that correspond to physically realizable measurements, in principle, and ones for which the limit s→∞ exists for physically meaningful operators. Physical states form a subspace ${𝚿}_{\mathbb{P}}$ of the total union space 𝚿 which we represent as


(3.11) $$\begin{array}{lrr} & {𝚿}_{\mathbb{P}}\equiv \{|p\rangle :|p\rangle \in 𝚿,\langle p|{\hat{N}}^{m}|p\rangle <\infty \;\forall \;m\in \mathbb{N}\}\subset 𝚿 & \end{array}$$

where


(3.12) $$\begin{array}{lrr} & 𝚿\equiv \overset{\infty }{\underset{s=0}{⋃}}{𝚿}_{s}. & \end{array}$$

In the following we will also use a subscript ℙ on a function, such as $P_{\mathbb{P}}(\theta )$, to indicate that it has been derived for the class of states ${𝚿}_{\mathbb{P}}$, and also an operator such as ${\hat{Q}}_{\mathbb{P}}$ to indicate the property that ${\hat{Q}}_{\mathbb{P}}^{n}$ has the same matrix elements as ${\hat{Q}}^{n}$ for arbitrary powers n when acting on ${𝚿}_{\mathbb{P}}$ in the infinite-s limit.

The commutator between the photon number operator ${\hat{N}}_{s}\;$, which is defined on ${𝚿}_{s}$ by


(3.13) $$\begin{array}{lrr} & {\hat{N}}_{s}\equiv \sum_{n=0}^{s}n|n\rangle \langle n|, & \end{array}$$

and the Hermitian phase angle operator ${\hat{\phi }}_{\theta }$ reduces for physical states to


(3.14) $$[{\hat{\phi }}_{\theta }\;,{\hat{N}}_{s}{]}_{P}=-i[{\hat{1}}_{s}-(s+1)|\theta_{0}\rangle_{\;s}{,}_{s\;}\langle \theta_{0}|].$$

Equation (3.14) differs markedly from Dirac’s commutator $[\hat{\phi },\hat{N}]=-i\hat{1}$ in (2.3). Dirac derived his commutator using the commutator–Poisson bracket correspondence and a non-cyclic variable for the phase angle. That is, in Dirac’s theory, phase angles differing by multiples of 2π are different. This is a nonphysical characteristic of an isolated field mode for which one period is equivalent to any other. In the Pegg–Barnett formalism, however, the phase angle is cyclical due to the cyclic property of the phase states themselves, since from (3.2),


(3.15) $$\begin{array}{lcr} & |\theta_{m}+2n\pi \rangle_{s}=|\theta_{m}\rangle_{s}. & \end{array}$$

Indeed, using the Heisenberg equation of motion, the time derivative of the expectation value of the phase angle $\langle {\hat{\phi }}_{\theta }\rangle =\left\langle p|{\hat{\phi }}_{\theta }|p\right\rangle$ for the physical state $|p\rangle \in {𝚿}_{\mathbb{P}}$ is found to be


(3.16) $$\frac{d}{dt}\langle {\hat{\phi }}_{\theta }\rangle =i\langle p|[\omega \hat{N},{\hat{\phi }}_{\theta }{]}_{P}|p\rangle =-\omega +\omega 2\pi P_{P,s}(\theta_{0})$$

where ω is the frequency of the field mode, and


(3.17) $$\begin{array}{lrr} & P_{\mathbb{P},s}(\theta )\equiv \frac{s+1}{2\pi }|\langle p|\theta \rangle_{s}{|}^{2} & \end{array}$$

is the phase probability density normalized over a 2π interval for a physical state |p⟩, and $|\theta \rangle_{s}$ is a general phase state in (3.1). Over a short duration of δt, the first term on the right side of (3.16) tends to reduce $\left\langle {\hat{\phi }}_{\theta }\right\rangle$ by ωδt whereas the second term tends to increase it by an amount that is proportional to the chance the phase is passing through the reference value $\theta_{0}$. The increases accumulate to 2π over a period and this ensures $\theta_{0}\leq \langle {\hat{\phi }}_{\theta }\rangle <\theta_{0}+2\pi$. The departure of the photon number-phase commutator in the Pegg-Barnett formalism in (3.14) from the so-called canonical commutator^5^ (2.3) is, therefore, due to the cyclic nature of the phase observable. We will say more about this later in §5.

An ‘acid test’ [24] for any theoretical model of phase, due to Barnett and Pegg [12,13,25], is that any 2π-periodic phase probability density P(θ) that yields a mean phase θ that is invariant to an arbitrary phase shift α, i.e. $\bar{\theta }=\int_{2\pi }\;\theta P(\theta +\alpha )d\theta$, is necessarily uniform, i.e. $P(\theta )=\frac{1}{2\pi }$ for all θ, and yields a phase variance of $\frac{\pi^{2}}{3}$. They also argue from a purely operational perspective that inserting a time delay in the path of a travelling optical field is equivalent to inducing a corresponding phase shift. Further, as each photon number state is a stationary state, its phase probability density is invariant to such time delays and corresponding phase shifts. It follows that for all photon number states, including the vacuum, any physically-legitimate phase probability density should be uniform and so yield a phase variance of $\frac{\pi^{2}}{3}$.

It is clear that Pegg and Barnett’s phase probability density in (3.17) passes the test. Indeed,


$$P_{P}(\theta )=\frac{1}{2\pi },$$

where


(3.18) $$P_{P}(\theta )\equiv \lim_{s\to \infty }P_{P,s}(\theta )$$

with $P_{P,s}(\theta )$ from (3.17), for the photon number states |n⟩ for n=0,1,…, and so each one has a uniform phase distribution and, thus, a purely random phase. This result follows from the deliberately engineered, mutually unbiased relation between the number basis {|n⟩:n=0,1,…,s} and the phase basis $\left\{|\theta_{m}\rangle_{s}:m=0,1,\ldots ,s\right\}$ in ${𝚿}_{s}$, where unbiased refers to the fact that $\langle n|\theta_{m}\rangle_{s}=(s+1{)}^{-1/2}$ is independent of n and m [13].

In addition to the number phase commutator in (3.14) there are also commutators between the number operator and the cosine and sine phase operators to consider. All have special states which give limited uncertainty in the operators, and because the commutators are non-trivial operators, they fall into two classes called *intelligent states* and regular *minimum uncertainty states*.^6^ We briefly mention those that are members of the physical states ${𝚿}_{\mathbb{P}}$. The number state |n⟩ is found to be the only exact intelligent state and minimum uncertainty state for all commutators. There are, however, approximate intelligent states which have approximately Gaussian probability distributions for both photon number and phase. Further details can be found elsewhere [37].

The creation and annihilation operators acting on ${𝚿}_{s}$ are defined in terms of the unitary phase operators and the number operator as


(3.19) $$\begin{array}{rl} {\hat{a}}_{s} & \equiv e^{i{\hat{\phi }}_{\theta }}{\hat{N}}_{s}^{1/2}=|0\rangle \langle 1|+2^{1/2}|1\rangle \langle 2|+\ldots +s^{1/2}|s-1\rangle \langle s|, \end{array}$$

(3.20) $$\begin{array}{rl} {\hat{a}}_{s}^{†} & \equiv {\hat{N}}_{s}^{1/2}e^{-i{\hat{\phi }}_{\theta }}. \end{array}$$

Equation (3.19) and (3.20) express ${\hat{a}}_{s}$ and ${\hat{a}}_{s}^{†}$ in terms of a unitary operator and a Hermitian one (${\hat{N}}^{1/2}$). Moreover, these expressions represent the polar decomposition on $\Psi_{s}\;$, following the approach suggested by Dirac and first attempted by Susskind and Glogower on 𝐇. The action of these operators mimics that of the conventional creation and annihilation operators if our state of interest is orthogonal to highly excited number states |n⟩ with n∼s. The commutation relation can be found in (3.5), (3.19) and (3.20),


(3.21) $$[{\hat{a}}_{s}\;,{\hat{a}}_{s}^{†}]={\hat{1}}_{s}-(s+1)|s\rangle \langle s|.$$

The trace of this commutator vanishes as it must for a finite-dimensional space. The term −(s+1)|s⟩⟨s| on the right side of (3.21), which results from the cyclical nature of the ladder operators $e^{i{\hat{\phi }}_{\theta }}$ and $e^{-i{\hat{\phi }}_{\theta }}$ on ${𝚿}_{s}$, is missing from the corresponding commutator $[\hat{a},{\hat{a}}^{†}]=\hat{𝟙}$ on 𝐇, however, it has no effect when acting on physical states. That is, the matrix elements of arbitrary powers n of the commutator, i.e.


(3.22) $$\begin{array}{lcr} & \left\langle p^{\prime }|[{\hat{a}}_{s},{\hat{a}}_{s}^{†}{]}^{n}|p\rangle =\langle p^{\prime }|\left[({\hat{𝟙}}_{s}-|s\rangle \langle s|)+(-1{)}^{n}(s+1{)}^{n}|s\rangle \langle s|\right]|p\right\rangle & \end{array}$$

tends to unity in the infinite-s limit for physical states $|p\rangle ,|p^{\prime }\rangle \in {𝚿}_{\mathbb{P}}$ and so, *formally*, the commutator


(3.23) $$\begin{array}{lcr} & [\hat{a_{s}},{\hat{a_{s}}}^{†}{]}_{\mathbb{P}}={\hat{𝟙}}_{s} & \end{array}$$

is all that is needed when dealing with physical states ${𝚿}_{\mathbb{P}}$. Thus, the difficulties associated with the Susskind–Glogower operators are elegantly circumvented with Pegg–Barnett’s use of their limiting procedure and restriction to physical states; more will be said on this in §3(c).

### (b) Infinite dimensional forms

Restricting the s→∞ limit exclusively to expectation values means all other calculations involving states and operators are performed with finite-dimensional objects in ${𝚿}_{s}$. This raises the question of the status of those objects in the infinite-s limit. For this we need to consider the two types of convergence, *strong* and *weak*, for sequences of vectors and operators on a normed vector space. A brief overview of convergence is given in Appendix A(b).

The total union space 𝚿 in (3.12) is not equipped with a norm [9]. Even if we define a suitable norm for it using, say, the norm defined on 𝐇, the limit points of Cauchy sequences would not necessarily belong to 𝚿. In contrast, 𝐇 is closed by definition, and so it contains the limit points of all its Cauchy sequences. The sequences converge strongly in this case. Further, for weak convergence, the limit points of Cauchy sequences are not necessarily in either 𝚿 or 𝐇, and additional care needs to be taken. We shall return to the issue of the space containing the limit points of weakly convergent sequences in the next subsection.

Any vector |ψ⟩∈𝐇 can be represented in ${𝚿}_{s}$ for every value of s by its projection^7^ $|\psi \rangle_{s}={\hat{𝟙}}_{s}|\psi \rangle$ using the identity operator ${\hat{𝟙}}_{s}$ given in (3.9). The sequence $|\psi \rangle_{s}$ for s=0,1,… necessarily converges strongly in 𝐇 owing to the completeness of 𝐇 [41]. A similar situation holds for bounded operators $\hat{Q}$ on 𝐇 and their projections ${\hat{Q}}_{s}\equiv {\hat{𝟙}}_{s}\hat{Q}{\hat{𝟙}}_{s}$ onto ${𝚿}_{s}$: the sequence ${\hat{Q}}_{s}$ for s=0,1,… converges strongly on 𝐇 for the same reason [41].

The situation is quite different when considering states and operators associated with phase as their definitions are specific for each value of s. Additionally, when considering a sequence of Pegg–Barnett phase states $|\theta_{m}\rangle_{s}$ in (3.2) for s=0,1,…, we need to decide on the value of m to use for $\theta_{m}$ in (3.3) for each value of s. If the value of m is fixed as s increases, then the value of $\theta_{m}$ will tend to $\theta_{0}$ as s increases and we will not be able to explore phase angles in a full 2π range. To avoid this, we first choose the phase value θ in the range $\theta_{0}\leq \theta <\theta_{0}+2\pi$ that we want to approach in the s→∞ limit, and then let the value of m in (3.3) be given uniquely by $m_{s}$ in $\theta \leq \theta_{m_{s}}<\theta +2\pi /(s+1)$. There is an uncountable number of choices for θ in this approach. As $\||\theta_{m_{s}}\rangle_{s}-|\theta_{m_{r}}\rangle_{r}{\|}^{2}>2(1-\sqrt{\left(r+1)/(s+1\right)})$ using the reverse triangle inequality |x−y|≥||x|−|y||, we find the sequence $|\theta_{m_{s}}\rangle_{s}$ for s=0,1,… fails to converge strongly in the same way that the implicit sequence of Loudon phase states $|\phi \rangle_{s}$ in (2.9) fails.

The phase states do converge weakly, however, on the space of physical states. This fact underlies the definition of the phase probability distribution in (3.17), (3.18). That is, the sequence of (scaled) inner products $\sqrt{s+1}\langle p|\theta_{m_{s}}\rangle_{s}=\sum_{n=0}^{s}\;p_{n}^{*}e^{in\theta_{m_{s}}}$ for s=0,1,… with $|p\rangle \in {𝚿}_{\mathbb{P}}$ converges to $\langle p|\theta \rangle =\sum_{n=0}^{\infty }\;p_{n}^{*}e^{in\theta }$ where |θ⟩ is the Susskind-Glogower phase state in (2.5). In short, the Pegg–Barnett phase states converge weakly to Susskind-Glogower phase states, i.e. $\sqrt{s+1}|\theta_{m_{s}}\rangle_{s}\overset{w}{⟶}|\theta \rangle$. The limit implicit in (2.5) is valid only in this weaker sense. Although |θ⟩ is said to be a weak limit, it is not valid to use it as a vector because the actual limit point is an inner product with physical states, and so its use is restricted to being in that form only.

We are led, therefore, to think of the infinite-s form of $|\theta_{m_{s}}\rangle_{s}$ as a *generalized state* analogously to the way we think of the infinitesimal-σ form of the Gaussian $g_{\sigma }(x)\equiv \frac{1}{\sigma \sqrt{2\pi }}e^{-x^{2}/2\sigma^{2}}$ as a generalized function (i.e. as the Dirac-δ distribution δ(x)). Recall that the limit σ→0 is taken not of $g_{\sigma }(x)$ itself but of integrals such as $\int g_{\sigma }(x)p(x)dx$ where p(x) is a test function that is differentiable to all orders. Likewise, the s→∞ limit is not taken of $|\theta_{m_{s}}\rangle_{s}$ but of inner products such as $\langle p|\theta_{m_{s}}\rangle_{s}$ with physical states |p⟩ for which the energy moments are finite to all orders. This comparison leaves us with no escape from viewing the infinite-s form of $|\theta_{m_{s}}\rangle_{s}$ in terms of the rigged Hilbert space structure. We will also pick up this thread in the next section.

Finally, it immediately follows from $|\theta_{m_{s}}\rangle_{s}\overset{w}{⟶}|\theta \rangle$ and the product law for limits that the phase probability density in (3.17), (3.18) satisfies


(3.24) $$\begin{array}{r} P_{P,s}(\theta )=\lim_{s\to \infty }P_{P,s}(\theta )=\frac{1}{2\pi }\langle \theta |p\rangle \langle p|\theta \rangle \end{array}$$

where |θ⟩ is the Susskind–Glogower phase state in (2.5). The operator associated with the right side is called the probability operator measure (POM) for physical states. Specifically, the continuous phase POM is defined as the set $\left\{d\hat{\Pi }(\theta )\right\}$ with $\int_{2\pi }d\hat{\Pi }(\theta )=\hat{1}$ where


(3.25) $$d\hat{\Pi }(\theta )\equiv \frac{1}{2\pi }|\theta \rangle \langle \theta |d\theta ,$$

|θ⟩ is the Susskind–Glogower phase state in (2.5), and


(3.26) $$\begin{array}{rl} P_{P}(\theta )d\theta & \equiv \langle d\hat{\Pi }(\theta )\rangle . \end{array}$$

The convergence of the Pegg–Barnett phase operators is similar to that for the phase states. The operators ${\hat{\phi }}_{\theta }$, $e^{i{\hat{\phi }}_{\theta }}$ and $e^{-i{\hat{\phi }}_{\theta }}$ do not converge strongly on 𝐇, but they do converge weakly on the physical states [41]. The weak limits of the exponential phase operators $e^{i{\hat{\phi }}_{\theta }}$ and $e^{-i{\hat{\phi }}_{\theta }}$ are the corresponding Susskind–Glogower operators ${\hat{e}}^{i\phi }$ and ${\hat{e}}^{-i\phi }$. But the weak limits do not necessarily preserve the algebra of the operators on ${𝚿}_{s}$—this is a general property of the weak limits of operators. Significantly, $e^{i{\hat{\phi }}_{\theta }}e^{-i{\hat{\phi }}_{\theta }}$$=e^{-i{\hat{\phi }}_{\theta }}e^{i{\hat{\phi }}_{\theta }}={\hat{1}}_{s}$ on ${𝚿}_{s}$ whereas we have already seen that ${\hat{e}}^{i\phi }{\hat{e}}^{-i\phi }=\hat{1}$ and ${\hat{e}}^{-i\phi }{\hat{e}}^{i\phi }=\hat{1}-|0\rangle \langle 0|$. In spite of this, the algebra on ${𝚿}_{s}$ can be recovered for the weak limits by adopting the *antinormal ordering* of the phase operators, defined as follows [42–44]. Any function of θ that has a Fourier series such as $f(\theta )=\sum_{k}\;f_{k}e^{ik\theta }$, where k ranges over the integers, is represented as the Pegg–Barnett operator $f({\hat{\phi }}_{\theta })=\sum_{k}\;f_{k}e^{ik{\hat{\phi }}_{\theta }}$ on ${𝚿}_{s}$. The weak limit is given by $f({\hat{\phi }}_{\theta })\overset{w}{⟶}\sum_{k}\;f_{k}{\hat{e}}^{ik\phi }\equiv \hat{f}(\phi )$ where ${\hat{e}}^{ik\phi }\equiv ({\hat{e}}^{i\phi }{)}^{k}$ for k≥0 and ${\hat{e}}^{ik\phi }\equiv ({\hat{\;e}}^{-i\phi }{)}^{|k|}$ for k<0. The antinormal order of arbitrary products of any number of factors of ${\hat{e}}^{i\phi }$ and ${\hat{\;e}}^{-\;i\phi }$ is defined to be with all factors of ${\hat{e}}^{i\phi }$ to the left of all factors of ${\hat{e}}^{-i\phi }$, and the operation of antinormal-ordering an arbitrary expression X is written with special brackets ${,}_{*}^{*}X{,}_{*}^{*}$. Thus, e.g. e∗∗e^iϕe^−iϕe∗∗=e^iϕe^−iϕ=1^ and $e_{*}^{*}{\hat{e}}^{-i\phi }{\hat{e}}^{i\phi }e_{*}^{*}={\hat{e}}^{i\phi }{\hat{e}}^{-i\phi }=\hat{1}$, which demonstrates that the Susskind–Glogower operators are essentially *unitary* under the antinormal ordering. More generally, $f({\hat{\phi }}_{\theta })g({\hat{\phi }}_{\theta })h({\hat{\phi }}_{\theta })\;\cdots \overset{w}{⟶}e_{*}^{*}\hat{f}(\phi )\hat{g}(\phi )\hat{h}(\phi )\;\cdots e_{*}^{*}$ where f(θ), g(θ) and h(θ), and so on, are arbitrary functions of θ.

There is also a way to construct phase operators that, like the Susskind–Glogower operators, are of infinite rank, but have the advantage of faithfully retaining the algebra of the Pegg–Barnett operators. Vaccaro and Bonner showed [45] in 1995 that the Pegg–Barnett phase formalism essentially builds an infinite-dimensional linear vector space 𝐄⊃𝐇 containing vectors in the form of *sequences* such as $|v\rangle =(|v\rangle_{0},|v\rangle_{1},|v\rangle_{2},\ldots \;)$ where $|v\rangle_{s}$ belongs to ${𝚿}_{s}$, and on which the operators are also *sequences* such as $\hat{Q}=\left({\hat{Q}}_{0},{\hat{Q}}_{1},{\hat{Q}}_{2},\ldots \;\right)$ where ${\hat{Q}}_{s}$ is an operator on ${𝚿}_{s}$. Expectation values are correspondingly given by $\langle v|\hat{Q}|v\rangle =\lim_{s\to \infty }\left({,}_{s}\langle v|{\hat{A}}_{s}|v\rangle_{s}/{,}_{s}\langle v|v\rangle_{s}\right)$. The phase operators ${\hat{\phi }}_{\theta }$, $e^{i{\hat{\phi }}_{\theta }}$ and $e^{-i{\hat{\phi }}_{\theta }}$ are faithfully represented as infinite-rank operators on 𝐄.

Yet another infinite dimensional form was given by one of us (JAV) [46] by constructing a Hilbert space ${𝐇}_{\mathrm{sym}}$ based on the closure of 𝚿 in (3.12) but where the relationship between the subspaces ${𝚿}_{s}$ for increasing s is of a particular symmetric kind. Consider an arbitrary, infinite orthonormal basis from which we choose the basis of each of the vector spaces ${𝚿}_{s}$. By judicious labelling, the first s and the last s states of the basis of ${𝚿}_{2s-1}$ are common, respectively, with the first s and the last s states of the basis of ${𝚿}_{2s+1}$. The two extra basis states unique to ${𝚿}_{2s+1}$ are in the middle of its basis set. We identify the first s basis states of ${𝚿}_{2s-1}$ with the basis of the conventional Hilbert space 𝐇, and the remaining s basis states with another Hilbert space $\tilde{𝐇}$ that is disjoint from 𝐇. This results in $𝐇\subset {𝐇}_{\mathrm{sym}}⊃\tilde{𝐇}$. As the exponential phase operator $e^{i{\hat{\phi }}_{\theta }}$ in (3.5) maps the lowest number state in ${𝚿}_{s}$ to its highest number state, this corresponds on ${𝐇}_{\mathrm{sym}}$ to mapping the vacuum state of 𝐇 to the ‘inverted vacuum’ state of $\tilde{𝐇}$. This property is a crucial one because it leads to the operators ${\hat{\phi }}_{\theta }$, $e^{i{\hat{\phi }}_{\theta }}$ and $e^{-i{\hat{\phi }}_{\theta }}$ converging *strongly* on ${𝐇}_{\mathrm{sym}}$. Full details can be found in [46]. Ozawa used the theory of non-standard numbers to build essentially the same construction [47].

### (c) Relation to other attempts

Pegg and Barnett’s acid test provides a physically motivated means of assessing attempts to define quantum phase. It amounts to the requirement that for all the photon number states (including the vacuum) any legitimate phase probability density should be uniform and so yield a variance in phase angle of $\frac{\pi^{2}}{3}$. We have seen in §2 that the Susskind–Glogower phase operators attribute the vacuum as having a preference for some phase angles over others, which clearly fails the test. Moreover, we have seen how the Susskind–Glogower phase operators are weak limits of the corresponding Pegg–Barnett operators, and also that weak limits do not necessarily preserve the algebra of the sequences [25]. Popov and Yarunin [34–36] (see also [48,49]) considered an equivalent set of phase eigenstates to that given in (3.2), (3.3) and constructed equivalent phase operators on a finite-dimensional space. However, they *insisted* that the limit s→∞ be taken of the operators, resulting in a description involving weak limits that is equivalent to that of Susskind and Glogower.

In 1991, Shapiro and Shepard constructed the phase POM in (3.25) using the Susskind–Glogower phase states and used it to derive the same phase probability density $P_{\mathbb{P}}(\theta )$ as in (3.26) to estimate a phase shift in an optical field. The probability density passes the acid test, and expectation values of functions of phase, such as M(θ), calculated using their probability density, i.e. $\langle M(\theta )\rangle =\int M(\theta )P_{\mathbb{P}}(\theta )\;d\theta$, gives results consistent with the Pegg–Barnett formalism. The reason for this success is the fact that $\int M(\theta )P_{P}(\theta )d\theta =\langle \int M(\theta )d\hat{\Pi }(\theta )\rangle =\langle \int \frac{1}{2\pi }M(\theta )|\theta \rangle \langle \theta |d\theta \rangle$ and $\int \frac{1}{2\pi }M(\theta )|\theta \rangle \langle \theta |d\theta ={†}_{†}^{†}\hat{M(\phi )}{†}_{†}^{†}$ [35], and so $\langle M(\theta )\rangle =\left\langle {,}_{*}^{*}\hat{M(\phi )}{,}_{*}^{*}\right\rangle$ is the expectation value of the antinormally-ordered version of the equivalent Susskind–Glogower function $\hat{M(\phi )}$. So although they based their analysis on the Susskind–Glogower approach, their fortuitous use of the probability density $P_{\mathbb{P}}(\theta )$ with a classical phase variable M(θ) ended up correct.

Finally, optical phase is intrinsically a relational property due to the fact that optical fields are subject to a global U(1) symmetry that results from the conservation of energy, and so it has physical meaning only relative to the phase of another field. This has been used as grounds for abandoning the quantum description of the phase of a single field and using the phase difference between two optical fields in its place. The argument fails, however, because there exist useful quantum descriptions of other relational properties. For example, position is also a relational property because the conservation of momentum manifests as the invariance of physical laws to global spatial translations and so absolute position is physically meaningless, but in this case, there is a well-behaved position operator that has undeniable conceptual relevance in conventional quantum mechanics. There is no need, therefore, to abandon the phase of a single field. On the contrary, the success of the Pegg–Barnett formalism means that the difference in the phases of two fields follows naturally from the phases of each [50,51]. Nevertheless, there have been attempts to give alternative definitions [52–54].

## 4. Construction of an enlarged rigged Hilbert space

We turn now to the relation between the rigged Hilbert space and Pegg and Barnett’s phase formalism, and this raises the issue of the topology of various vector spaces. Topology refers to linearity, continuity and convergence properties [7], and as linearity and continuity are the usual assumptions for vector spaces in quantum mechanics, the issue for us falls to the way convergence is defined for sequences of vectors. As convergence is explicitly required for sequences of expectation values in Pegg and Barnett’s formalism and not state vectors, there may appear at first glance to be no need for any topology, as Pegg and Barnett have suggested [9]. However, the physical states $|p\rangle \in {𝚿}_{\mathbb{P}}$ in (3.11) are a key component of the calculation of expectation values, and they do require the partial sums $\sum_{n=0}^{s}|p_{n}|n^{m}$ to converge to the limit point $\left\langle p|{\hat{N}}^{m}|p\right\rangle$ as s→∞. The role played by physical states, therefore, calls for closer inspection.

The family of physical states ${𝚿}_{\mathbb{P}}$ is unchanged if the condition for membership is modified from $\langle p|{\hat{N}}^{m}|p\rangle <\infty$ to $\langle p|(\hat{N}+1{)}^{m}|p\rangle <\infty$ for any positive integer m. As it is more convenient, we will use the modified version. The value of $\left\langle p|(\hat{N}+1{)}^{m}|p\right\rangle$ for state |p⟩ fulfils the requirements^8^ for being a norm of |p⟩ which we shall write as $\||p\rangle {\|}_{m}\equiv \langle p|(\hat{N}+1{)}^{m}|p\rangle$ and refer to as the m th *moment* norm. The physical state |p⟩∈𝐇 is the limit point of the Cauchy sequence $|p\rangle_{s}\in {𝚿}_{s}\subset 𝚿$ for s=0,1… for the m th moment norm for any given positive integer value of m where $|p\rangle_{s}={\hat{𝟙}}_{s}|p\rangle$ is the projection of |p⟩ onto ${𝚿}_{s}$. Such limit points, however, are not necessarily elements of ${𝚿}_{\mathbb{P}}$ or 𝚿, neither of which has a topology. The closure given by the union of ${𝚿}_{\mathbb{P}}$ with these limit points is the vector space 𝚽 in the relation $𝚽\subset 𝐇\subset {𝚽}^{\prime }$ in (2.8) for the rigged Hilbert space [7]. The topology of 𝚽 is its linearity, continuity and Cauchy convergence for all moment norms.

As demonstrated in the previous subsection, the weak limit of the phase eigenstates, $\sqrt{s+1}|\theta_{m_{s}}\rangle_{s}\overset{w}{⟶}|\theta \rangle$, is defined in terms of an inner product with any physical state $|p\rangle \in {𝚿}_{\mathbb{P}}$, i.e. it is defined on the space ${𝚿}_{\mathbb{P}}$, and as 𝚽 is the closure of ${𝚿}_{\mathbb{P}}$, the limit is automatically defined on 𝚽. This means that the phase eigenstates are in a dual relation with the $|p\rangle \in {𝚿}_{\mathbb{P}}$, and so automatically the weak limit |θ⟩ is an element of ${𝚽}^{\prime }$ because ${𝚽}^{\prime }$ is the dual space of 𝚽. The phase POM in (3.25) is, accordingly, defined in terms of ${𝚽}^{\prime }$.

So although 𝚽 and ${𝚽}^{\prime }$ are not *explicit* components of the Pegg–Barnett formalism, they are *all but constructed* in parallel with the limit points of sequences of expectation values. Even so, there is no necessity to use the rigged Hilbert space structure for quantum phase, however, if it is used it brings Pegg–Barnett’s description of quantum phase into line with the treatment of Dirac’s δ-distribution. Recall that the δ-distribution is defined in terms of an integral of its product with a smooth test function belonging to Schwartz space ${𝚽}_{\tau }$. It is straightforward to show that 𝚽 is isomorphic to ${𝚽}_{\tau }$ with the mapping $𝚽∋|p\rangle \mapsto p(\theta )\in {𝚽}_{\tau }$ given by $p(\theta )=\frac{1}{\sqrt{2\pi }}\sum_{n=0}^{\infty }\;e^{in\theta }p_{n}$ where $p_{n}=\left\langle n|p\right\rangle$. The condition $\langle p|{\hat{N}}^{m}|p\rangle <\infty$ is equivalent to requiring the m th derivative of p(θ) to exist. In other words, a quantum mechanical treatment that makes use of the δ-distribution already is making use of the rigged Hilbert space represented by the relation $𝚽\subset 𝐇\subset {𝚽}^{\prime }$ that supports the Pegg–Barnett description of quantum phase provided the antinormal ordering reviewed in the previous subsection is adopted for the phase operators.

The caveat on antinormal ordering can be dispensed with if the Hilbert space 𝐇 is exchanged with ${𝐇}_{\mathrm{sym}}$ for then the phase operators are faithfully represented as strong limits. This brings us to the *minimal structure* necessary for the state space of quantum mechanics for representing observables as ordinary operators (i.e. as strong limits without special ordering) and all states (including position, momentum and phase eigenstates) of systems such as a free particle and a harmonic oscillator:


(4.1) $$\begin{array}{lrr} & 𝚽\subset {𝐇}_{\mathrm{sym}}\subset {𝚽}_{\mathrm{sym}}^{\prime } & \end{array}$$

where the dual to the space of physical states is now ${𝚽}_{\mathrm{sym}}^{\prime }⊃{𝚽}^{\prime }$ which is larger due to the additional possibilities allowed by the enlarged basis of ${𝐇}_{\mathrm{sym}}$ in comparison to that of 𝐇.

## 5. Defining principle: canonical conjugacy or complementarity?

The confusion surrounding the search for a generally acceptable quantum phase description is at least partly because, historically, quantum mechanics has been constructed using an imprecise analogy with classical mechanics based on Dirac’s commutator–Poisson bracket correspondence [27]. Briefly, in Hamiltonian mechanics a set of generalized coordinates $𝐪\equiv \left\{q_{i}\right\}$ are used to define a corresponding set of conjugate momenta $𝐩\equiv \left\{p_{i}\right\}$ by $p_{i}\equiv \partial L(𝐪,\dot{𝐪},t)/\partial {\dot{q}}_{i}$ where ${\dot{q}}_{i}$ is the time derivative of $q_{i}$ and $L(𝐪,\dot{𝐪},t)$ is the Lagrangian [55]. The variables $q_{i},p_{i}$ are known as a canonically conjugate pair and they satisfy $\{q_{i},p_{i}\}=1$ where $\{a,b\}\equiv \sum_{i}\frac{\partial a}{\partial q_{i}}\frac{\partial b}{\partial p_{i}}-\frac{\partial b}{\partial q_{i}}\frac{\partial a}{\partial p_{i}}$ is the Poisson bracket of a and b. Dirac’s prescription for constructing quantum mechanics is that an arbitrary pair of dynamical variables a=a(𝐪,𝐩), b=b(𝐪,𝐩) in Hamiltonian mechanics is replaced in quantum mechanics by operator equivalents $\hat{a},\hat{b}$ that satisfy the commutation relation $[\hat{a},\hat{b}]=\;i\hbar f(\hat{𝐪},\hat{𝐩})$ where f(𝐪,𝐩)={a,b} is the Poisson bracket expressed as a function of the canonically conjugate variables [27]. However, the mappings $a(q,p)\mapsto \hat{a}$, $b(𝐪,𝐩)\mapsto \hat{b}$, and $f(𝐪,𝐩)\mapsto f(\hat{𝐪},\hat{𝐩})$ are not unique, in general, due to the fact that the expressions for a(𝐪,𝐩), b(𝐪,𝐩), and f(𝐪,𝐩) are invariant to any reordering of products of the conjugate pairs $q_{i}$ and $p_{i}$, whereas any reordering products of ${\hat{q}}_{i}$ and ${\hat{p}}_{i}$ in each of $\hat{a}$, $\hat{b}$, and $f(\hat{𝐪},\hat{𝐩})$ is guaranteed to result in a change as $[{\hat{q}}_{i},{\hat{p}}_{i}]=\;i\hbar \{q_{i},p_{i}\}=\;i\hbar$. Moreover, all Poisson brackets are invariant under canonical transformations that take 𝐪 and 𝐩 to a new set of canonically conjugate pairs 𝐐=𝐐(𝐪,𝐩,t) and 𝐏=𝐏(𝐪,𝐩,t), which adds even more ambiguity. This problem, called the *Dirac problem*, has been long recognized and there are methods and theorems for dealing with only particular situations [56–58].

Crucially for us here, there is a canonical transformation for the harmonic oscillator from q and p to a different canonically conjugate pair, the action-angle variables J and ϑ, where J is defined by the integral $J\equiv \oint pdq=\frac{1}{\omega }2\pi \alpha$ over one cycle, ω is the angular frequency, α=H(q,p) is the total energy, and ϑ≡∂W/∂J where $W=\int p\dot{q}dt=\vartheta J$ is Hamilton’s characteristic function [55]. The transformed Hamiltonian is $H(J,\vartheta )=\frac{1}{2\pi }J\omega$ yielding Hamilton’s equations $\dot{\vartheta }=\frac{\partial }{\partial J}H(J,\vartheta )=\frac{1}{2\pi }\omega$ and $\dot{J}=-\frac{\partial }{\partial \vartheta }H(J,\vartheta )=0$. The angular solution is simply


(5.1) $$\begin{array}{lrr} & \vartheta (t)=\frac{1}{2\pi }\omega t+\beta & \end{array}$$

where β is a constant of integration, which shows that the angle increases indefinitely.

Dirac’s appeal to a canonically conjugate relation to arrive at (2.3) by the Poisson bracket-commutator correspondence is consistent with $J\mapsto \hat{N}h$ and $\vartheta \mapsto -\frac{1}{2\pi }\hat{\phi }$ and {ϑ,J}=1. While the fact that the phase angle ϑ in the classical description increases indefinitely in time is regarded as well-founded, there is an elemental difficulty associated with $\hat{\phi }$ having the same behaviour in the quantum description. This is because in quantum mechanics any Heisenberg picture observable $\hat{Q}(t)$ of the harmonic oscillator is invariant to a time shift of one period T, i.e. $\hat{Q}(T)=e^{i\hat{H}T/\hbar }\hat{Q}(0)e^{-i\hat{H}T/\hbar }=\hat{Q}(0)$ because the time evolution operator $e^{-i\hat{H}T/\hbar }=\hat{1}$ is trivial in that circumstance. The quantum phase observable, therefore, needs to be periodic and the non-periodic angle variable ϑ is a poor choice as its classical analogy. This conclusion represents a *physical basis* for the deficiency underpinning Dirac’s attempt in (2.3) and it supplements the mathematical one pointed out by Louisell [20].

Judge and Lewis [59] drew attention in 1963 to a better choice for the classical analogy: Y(ϑ)=ϑ modulo 2π where ϑ is given by (5.1). Pegg and Barnett [11] then showed in 1989 how their Hermitian phase operator ${\hat{\phi }}_{\theta }$ is related to an equivalent periodic function of ϑ given by


(5.2) $$\begin{array}{lrr} & \phi_{\theta }=\arctan(\frac{p}{\omega q}), & \end{array}$$

choosing the interval $\left[\theta_{0},\theta_{0}+2\pi \right)$ as the principle branch of the arctan function for a given reference phase $\theta_{0}$. The Poisson bracket with the harmonic oscillator Hamiltonian $H=\frac{1}{2}(p^{2}+\omega^{2}q^{2})$ for a unit mass can be written concisely in terms of $\phi_{\theta }$ as [11]


(5.3) $$\begin{array}{lrr} & \{\phi_{\theta },H\}=-\omega [1-2\pi \delta (\phi_{\theta }-\theta_{0})]. & \end{array}$$

The expectation value of $\left\{\phi_{\theta },H\right\}$ for an arbitrary classical phase probability density P(θ) normalized over any 2π interval, $\int_{2\pi }\;P(\theta )d\theta =1$, is given by


(5.4) $$\begin{array}{r} \bar{\left\{\phi_{\theta },H\right\}}=\int_{2\pi }P(\phi_{\theta })\{\phi_{\theta },H\}d\phi_{\theta }=-\omega [1-2\pi P(\theta_{0})]. \end{array}$$

Under Dirac’s correspondence principle, we need to compare this with the expectation value of $\left[{\hat{\phi }}_{\theta },\hat{H}]/(i\hbar \right)$. Using $\hat{H}=(\hat{N}+\frac{1}{2})\hbar \omega$ with the left side of (3.16) gives


(5.5) $$\begin{array}{r} \frac{1}{i\hbar }\langle p|[{\hat{\phi }}_{\theta }\;,\hat{H}]|p\rangle =-\omega [1-2\pi P_{P}(\theta_{0})] \end{array}$$

where $P_{\mathbb{P}}(\theta )$ is given by (3.17) and (3.18). Thus, Pegg and Barnett confirmed that Dirac’s commutator–Poisson bracket correspondence for the periodic function of phase is *precise* for physical states [11].

However, Pegg and Barnett did *not* derive their phase operator ${\hat{\phi }}_{\theta }$ from any commutator–Poisson bracket correspondence as we’ve already seen in §3(a). Instead, the success of their approach can be traced to their implicit use of mutually unbiased bases together with their new limiting procedure. Mutually unbiased bases were introduced by Schwinger [60] in 1960 with unitary operators on finite-dimensional Hilbert spaces. Two orthonormal bases $\{|u_{k}\rangle :k=1,2,\ldots ,D\}$ and $\{|v_{k}\rangle :k=1,2,\ldots ,D\}$ of a D dimensional vector space are said to be mutually unbiased if $|\langle u_{j}|v_{k}\rangle {|}^{2}=\frac{1}{D}$ for all j,k=1,2,…,D. Schwinger showed further that if the bases also satisfied an inner product relation of the form


(5.6) $$\begin{array}{r} \langle u_{j}|v_{k}\rangle =\frac{1}{\sqrt{D}}e^{i2\pi jk/D}, \end{array}$$

which is the kernel of the discrete Fourier transform, then the unitary operators constructed as


(5.7) $$\begin{array}{r} \hat{U}\equiv \sum_{k=1}^{D}e^{i2\pi k/D}|u_{k}\rangle \langle u_{k}|,\;\text{and}\;\hat{V}\equiv \sum_{k=1}^{D}e^{-i2\pi k/D}|v_{k}\rangle \langle v_{k}| \end{array}$$

cyclically permute the eigenstates of the other operator, i.e. $\hat{U}|v_{k}\rangle =|v_{k+1}\rangle$ and $\hat{V}|u_{k}\rangle =|u_{k+1}\rangle$ where $|u_{D+1}\rangle \equiv |u_{1}\rangle$ and $|v_{D+1}\rangle \equiv |v_{1}\rangle$. Moreover, he recognized $\hat{U}$ and $\hat{V}$ as being *maximally incompatible* operators in the sense that a projective measurement in the eigenbasis of one leaves no information about the outcome of a subsequent projective measurement in the eigenbasis of the other, and regarded them as being a *complementary pair of operators*. Finally, Schwinger asserted that the limit D→∞ for prime D leads to $\hat{U}$ and $\hat{V}$ having Hermitian generators in the form of the position and momentum operators and, in his footnote 3 [60], he conjectured that all possible types of physical degrees of freedom could be identified by this method.

There is an additional point to be made here that is not mentioned by Schwinger. Consider the Hermitian generators $\hat{u}=\sum_{k=1}^{D}k|u_{k}\rangle \langle u_{k}|$ and $\hat{v}=\sum_{k=1}^{D}k|v_{k}\rangle \langle v_{k}|$ associated with the operators $\hat{U}=e^{i\hat{u}2\pi /D}$ and $\hat{V}=e^{i\hat{v}2\pi /D}$ in (5.7). Note that it is a matter of semantics whether we refer to the unitary operators $\hat{U}$ and $\hat{V}$, or their corresponding Hermitian generators, $\hat{u}$ and $\hat{v}$, as the complementary pair. As $\hat{u}$ generates (cyclical) translations in $\hat{v}$, i.e. $\hat{U}\hat{v}{\hat{U}}^{-1}=\sum_{k=2}^{D}\;(k-1)|v_{k}\rangle \langle v_{k}|+D|v_{1}\rangle \langle v_{1}|$, whereas it leaves $\hat{u}$ invariant, i.e. $\hat{U}\hat{u}{\hat{U}}^{-1}=\hat{u}$, it follows that the operator that is complementary to $\hat{u}$ is the one that $\hat{u}$ generates translations in, and correspondingly for $\hat{v}$. This means we can identify the complementary property as the one being translated by $\hat{u}$. Moreover, owing to its invariance to translations generated by $\hat{u}$, $\hat{u}$ has none of the property that is complementary to it. The complementary properties are *mutually exclusive* in this sense.

The mutually unbiased nature of the number {|n⟩:n=0,1,…,s} and phase eigenbases $\{|\theta_{m}\rangle :m=0,1,\ldots ,s\}$ in Pegg and Barnett’s formalism is clearly evident from (3.2). Moreover, the inner product relation for $\theta_{0}=0$,


(5.8) $$\begin{array}{r} \langle n|\theta_{m}\rangle_{s}=\frac{1}{\sqrt{s+1}}e^{i2\pi nm/(s+1)}, \end{array}$$

is in the same form as (5.6). (For convenience, we shall treat the special case where $\theta_{0}=0$; the extension to the general case $\theta_{0}\neq 0$ is, however, straightforward.) The unitary operators corresponding to $\hat{U}$ and $\hat{V}$ in (5.7) are $e^{i2\pi \hat{N}/(s+1)}$ and $e^{-i{\hat{\phi }}_{\theta }}$, respectively, where $e^{-i{\hat{\phi }}_{\theta }}$ is the Hermitian conjugate of the operator defined in (3.5). Moreover, $e^{i2\pi \hat{N}/(s+1)}$ and $e^{-i{\hat{\phi }}_{\theta }}$ cyclically permutes the eigenstates of the other operator, e.g. $e^{i2\pi \hat{N}/(s+1)}|\theta_{m}\rangle =|\theta_{m+1}\rangle$ with $|\theta_{s+1}\rangle =|\theta_{0}\rangle$ due to $\theta_{s+1}=\theta_{0}+2\pi$, and $e^{-i{\hat{\phi }}_{\theta }}$ cyclically permutes the photon number states as demonstrated above in (3.10). The operators are therefore, according to Schwinger [60], a complementary pair.

In 1990 Pegg *et al*. [61] showed that all three complementary operator pairs $\left(\hat{q},\hat{p}\right)$, $\left(\hat{N},{\hat{\phi }}_{\theta }\right)$, and $\left({\hat{L}}_{z},{\hat{\phi }}_{\theta }\right)$, can be constructed on the subspace of physical states, ${𝚿}_{\mathbb{P}}$ in (3.11), in the infinite-dimensional limit s→∞ simply by altering the details of the spectra of the two Hermitian generators and identifying corresponding sets of physical states. In particular, if both spectra are continuous and unbounded, the complementary pair are the position and momentum operators $\left(\hat{q},\hat{p}\right)$, if one spectrum is discrete and bounded below and the other is continuous and bounded, the pair are the photon number and phase operators $\left(\hat{N},{\hat{\phi }}_{\theta }\right)$, or if one spectrum is discrete and unbounded and the other is continuous and bounded, the pair are the z-component of angular-momentum and angle operators $\left({\hat{L}}_{z},{\hat{\phi }}_{\theta }\right)$. In other words, one unified approach can construct all three complementary pairs without reference to Dirac’s commutator–Poisson bracket correspondence. Although the authors were not aware of Schwinger’s prior work at the time, this demonstration is in accord with Schwinger’s conjecture three decades earlier. It also reinforces the importance of the rigged Hilbert space in (4.1) as the minimal state space required for all three pairs.

This remarkable demonstration offers new insight into the very foundations of quantum mechanics. In his book *The principles of quantum mechanics* [27], Dirac first makes a case for representing the states and observables of a physical system using a Hilbert space and associated linear operators. To align the representation with classical mechanics, he then proposes the commutator–Poisson bracket correspondence as a way of identifying pairs of operators with their classical variable counterparts on the *basis of the role the variables play in classical mechanics*. But we have seen that this correspondence is problematical owing to its ambiguity associated with operator ordering. Schwinger, on the other hand, identifies pairs of linear operators on Hilbert space that are in extreme relationships of maximum incompatibility that he associates with complementarity. This is an inherently quantum mechanical classification of operator pairs. The maximal incompatibility of the operators implies that they will play extremal roles in quantum mechanics, and indeed, this is evident in the complementary nature of their physically accessible information. Moreover, Pegg *et al.* [61] have shown that classical variables can be identified with pairs of complementary operators based on corresponding ranges of variable values and operator eigenvalues. Placing the commutator of complementary operators in correspondence with a Poisson bracket, as Pegg *et al.* also did, recasts the canonical variables in classical mechanics as being those that are *maximally incompatible in quantum mechanics*. Crucially, this correspondence is a mapping *from* complementary pairs in quantum mechanics *to* canonical pairs in classical mechanics, as it ought to be given that classical mechanics is regarded as a large-scale approximation of quantum mechanics. Significantly, it does not suffer from the ambiguity associated with Dirac’s commutator–Poisson bracket correspondence.

There is a clear case, therefore, for using complementarity in the form of the operator pairs represented by Schwinger [60] in (5.6), (5.7), and applied by Pegg and Barnett in their general approach, as the basis for defining fundamental operators in quantum mechanics in place of Dirac’s classically-based canonical conjugacy. Following Schwinger, we refer to such operators as forming *complementary pairs* and regard the relationship between them as being mutually complementary. This gives a mathematical basis to Bohr’s broadly defined complementarity principle^9^ by nominating pairs of operators for which physically accessible information is complementary in the sense of being maximally incompatible.

## 6. Discussion and conclusion

The lack of a satisfactory quantum description of phase was a troubling issue in quantum mechanics for 60 years. The problem became particularly acute when the investigation of the (relative) phase of single-mode optical fields came within experimental expertise. We have seen how Loudon’s book *The quantum theory of light* provided a fresh perspective that led Pegg and Barnett to their quantum phase formalism in 1989. The last 30 years have seen formalism become better understood and widely adopted as a part of quantum mechanics that needs a special, but justified, limiting procedure.

Alongside this was Dirac’s δ-distribution that, like quantum phase, was seen as a necessary component of quantum theory from its inception but was problematical mathematically. The rigged Hilbert space represented by the relationship $𝚽\subset 𝐇\subset {𝚽}^{\prime }$ provided the eventual resolution making it unnecessary to explicitly take the limit of a sequence of representations of the δ-distribution because the definition of ${𝚽}^{\prime }$ includes a limiting procedure for dealing with the (essentially weak) limit of the representations. The opportunity to review the phase formalism here more than 30 years after its introduction has allowed us to take a broader view and show that the limiting procedure of the phase formalism all but constructs a rigged Hilbert space in parallel with it. In fact, 𝚽 essentially represents the physical states that are a key part of the Pegg–Barnett formalism in the s→∞ limit. Also, the phase eigenstates mimic Dirac’s δ-distribution in that neither belongs to representations of 𝐇 in their limiting forms and just as the δ-distribution is an element of ${𝚽}^{\prime }$, so too are distribution-like representations of the phase eigenstates. The last piece needed to complete the new rigging is a Hilbert space on which the Pegg–Barnett phase operators converge faithfully (i.e. as strong limits), and it is given by the extended Hilbert space ${𝐇}_{\mathrm{sym}}$ constructed by one of us (JAV) in 1995 [46]. This brings us to conclude that the *minimal structure* necessary for the state space of quantum mechanics for representing observables such as position, momentum, energy and phase, angular momentum and angle as ordinary operators, and corresponding states including position, momentum, energy and phase, angular momentum and angle eigenstates, is given by the *extended* rigged Hilbert space represented by the relationship


(6.1) $$\begin{array}{lrr} & 𝚽\subset {𝐇}_{\mathrm{sym}}\subset {𝚽}_{\mathrm{sym}}^{\prime } & \end{array}$$

where ${𝚽}_{\mathrm{sym}}^{\prime }$ is the corresponding extended space of distributions.

Undertaking this review has also revealed ramifications of Pegg and Barnett’s phase formalism that appear to have been left unappreciated. The fact that Pegg and Barnett’s special limiting procedure preserves the mutually unbiased nature of the phase and photon number eigenbases is a key element that underlies the success of their approach. While they made it clear that their intention in the formalism was not to implement Dirac’s commutator–Poisson bracket correspondence to define phase, they showed that the formalism gave results consistent with it [11]. This justified the use of the classically derived adjectives ‘canonical’ and ‘conjugate’ to describe photon number and phase operators [61]. But there has been something missing for three long decades because nothing has been identified as the principle underpinning the formalism to replace the Dirac correspondence principle. However, as mentioned in the previous section, Schwinger identified mutually unbiased bases with complementary pairs of fundamental operators [60]. It is appropriate, therefore, to say using Schwinger’s nomenclature, that Pegg and Barnett’s general approach is aimed at defining complementary pairs as the fundamental operators in quantum mechanics in place of Dirac’s correspondence principle. As classical mechanics is regarded as a large-scale approximation of quantum mechanics, identifying the fundamental operators in quantum mechanics on a quantum-mechanical basis (i.e. associated with mutually unbiased bases) is preferred compared with the classical basis underlying Dirac’s correspondence principle. This shift in focus represents a reworking of the very foundations of quantum mechanics and is perhaps the most profound consequence of Pegg and Barnett’s resolution of the quantum phase problem.

Finally, in addition to being a direct stimulus for the quantum phase formalism, the flow-on effect from Loudon’s book can be seen in Pegg’s description of relational time [14] and definition of the quantum age of a system [15]. It also continues in our present research on quantum time [16,17,63].

## Data Availability

This article has no additional data.

## Footnotes

^1^
Note that Dirac defined his phase operator $\phi_{r}$ as a time lapse, and here $\hat{\phi }=-2\pi \nu_{r}\phi_{r}$ is the corresponding angle, where $\nu_{r}$ is the frequency, and r indexes the field mode [4]. The negative sign reflects the modern convention where the phase angle decreases in time. Also, Dirac worked with both Heisenberg ($\theta_{r}$) and Schrödinger ($\phi_{r}$) picture versions of the phase where $\theta_{r}=e^{iE_{r}t/\hbar }\phi_{r}\;e^{-iE_{r}t/\hbar }$ and $E_{r}$ is the corresponding energy operator.

^2^
Nevertheless, there is still hope: the first undergraduate physics text to include the rigged Hilbert space appears to be by Ballentine in 1998 [30] and there has been some encouraging work by MacKay [8].

^3^
The same set of phase states was discussed by Popov and Yarunin [34] in 1973 in a Russian journal, and was bought to the attention of the wider physics community in 1992 [35,36]. We briefly discuss its relation to the Pegg-Barnett formalism at the end of this section.

^4^
This should be compared with the Susskind–Glogower situation where ${\hat{e}}^{i\phi }$ annihilates the vacuum state, i.e. ${\hat{e}}^{i\phi }|0\rangle =0$.

^5^
It is referred to as the *canonical* commutation relation because Dirac based it on a correspondence with the Poisson bracket for canonically conjugate variables [4]

^6^
Recall the Heisenberg–Robertson uncertainty relation $\langle \Delta {\hat{A}}^{2}\rangle \langle \Delta {\hat{B}}^{2}\rangle \geq \frac{1}{4}\langle \hat{C}\rangle^{2}$ where $\hat{A}$ and $\hat{B}$ are arbitrary non-commuting operators and $\hat{C}$ is given by $[\hat{A},\hat{B}]=\;i\hat{C}$ [38]. The states that give the equality in the uncertainty relation are called intelligent states (following Aragone *et al .*) [39,40] and those that also minimize the product $\left\langle \Delta {\hat{A}}^{2}\rangle \langle \Delta {\hat{B}}^{2}\right\rangle$ are called minimum uncertainty states.

^7^
A projection onto ${𝚿}_{s}$ was referred to as a 𝚿-restriction in Ref. [41]

^8^
A norm ‖⋅‖ satisfies ‖x+y‖≤‖x‖+‖y‖, ‖αx‖=|α|‖x‖ for all scalars α, and ‖x‖=0 iff x=0, for all x,y∈V.

^9^
Bohr summarized the principle in 1948 with 'the impossibility of any sharp separation between the behaviour of atomic objects and the interaction with the measuring instruments which serve to define the conditions under which the phenomena appear'. and '...evidence obtained under different experimental conditions cannot be comprehended within a single picture, but must be regarded as complementary in the sense that only the totality of the phenomena exhausts the possible information about the objects'. [62] Note that a single picture ceases to be inadequate if all relevant phenomena and experimental evidence under scrutiny are associated with commuting observables in quantum mechanics because the description would be relative to a fixed eigenbasis basis and so be equivalent to a classical one.

## Appendix A. Convergence and the rigged Hilbert space

We briefly outline different types of convergences and the structure of the rigged Hilbert space.

### A.1.Convergence

Recall the definition of convergence of a sequence of numbers $t_{n}$ for n=0,1,…. The sequence converges if it satisfies Cauchy’s criterion, namely for all ϵ>0 there exists an $N_{\epsilon }$ such that $|t_{m}-t_{n}|<\epsilon$ for all $m,n>N_{\epsilon }$ where |⋅| is the absolute value [5]. In that case it is said to be a Cauchy sequence. Moreover, a number t is the limit point if for all ϵ>0 there exists an $N_{\epsilon }$ such that $|t_{n}-t|<\epsilon$ for all $n>N_{\epsilon }$, and we write $t_{n}\to t$. The criterion for the convergence of a sequence of vectors in or operators on a vector space, however, depends on how the potential limit point is to be treated. There are two types of converges that we will consider here, strong and weak.

If the limit point is to be treated as a *bona fide* object in exactly the same way as the objects in the sequence, the criteria for the convergence needs to reflect this. Consider a sequence of states $|v\rangle_{n}\in 𝐕$ for n=0,1,… in vector space 𝐕 which is equipped with a norm ‖⋅‖. For the limit point to be another vector in 𝐕, the terms in the sequence need to become *closer* to each other further along the sequence, where closer is defined in terms of the norm. The criterion for this is Cauchy convergence, where for every ϵ>0 there exists $N_{\epsilon }$ such that $\||v\rangle_{n}-|v\rangle_{m}\|<\epsilon$ for all $n,m>N_{\epsilon }$. If this is satisfied, the limit point |v⟩ satisfies $\||v\rangle_{n}-|v\rangle \|<\epsilon$ for all $n>N_{\epsilon }$ and the sequence is said to converge *strongly* to |v⟩. We’ll use the notation $|v\rangle_{n}\overset{s}{⟶}|v\rangle$ as n→∞ on 𝐕 for strong convergence. The limit point can be used in the same way as other vectors in 𝐕, such as being the target of an operator on 𝐕. This is the usual type of convergence encountered in quantum physics.

Alternatively, some quantities are derived from states, such as the inner product $v_{n}(x)\equiv \langle x|v\rangle_{n}$ where $|v\rangle_{n},|x\rangle \in 𝐕$, that are needed in a limit form rather than the limits of underlying states. The criterion for convergence in this situation is that the terms in the sequence of derived quantities, $v_{n}(x)$ for n=0,1,… need to become closer to each other as n increases, where the norm is now simply the absolute value |⋅|. Again, this requires Cauchy convergence in the form that for every ϵ>0 there exists an $N_{\epsilon }$ such that $|v_{n}(x)-v_{m}(x)|<\epsilon$ for every $n,m>N_{\epsilon }$ and every |x⟩∈𝐕. If satisfied, we can refer to the sequence $v_{n}(x)$ for n=0,1,… as converging to a limit point v(x). Alternatively, we can also say that the underlying sequence of states $|v\rangle_{n}$ for n=0,1,… converges *weakly* to |v⟩ on 𝐕, in which case we would write $|v\rangle_{n}\overset{w}{⟶}|v\rangle$ as n→∞ on 𝐕. But note that it is not valid to use a weak limit point in the same way as the terms of the sequence such as, e.g. the target of an operator, because the convergence criterion used does not explicitly support it. Finally, if a sequence converges strongly, then it also converges weakly to the same limit point.

An analogous situation exists for sequences of operators. As an operator $\hat{Q}$ on 𝐕 is a mapping from the vector space 𝐕 onto itself, i.e. $\hat{Q}:𝐕\mapsto 𝐕$, the convergence of a sequence of operators reduces to the convergence of the results of the mappings by the sequence. Thus, a sequence of operators ${\hat{Q}}_{n}$ for n=0,1,… on a normed vector space 𝐕, maps |v⟩∈𝐕 to the corresponding sequence of vectors $|q(v)\rangle_{n}\equiv {\hat{Q}}_{n}|v\rangle$. If $|q(v)\rangle_{n}\overset{s}{⟶}|q(v)\rangle$ as n→∞ and |q(v)⟩∈𝐕 for every |v⟩∈𝐕 then ${\hat{Q}}_{n}$ converges *strongly* on 𝐕 to $\hat{Q}$ where $\hat{Q}|v\rangle =|q(v)\rangle$, and we write ${\hat{Q}}_{n}\overset{s}{⟶}\hat{Q}$ on 𝐕. Strong limit points preserve the algebra of the terms in the sequence, so if ${\hat{Q}}_{n}{\hat{P}}_{n}={\hat{R}}_{n}$, ${\hat{Q}}_{n}\overset{s}{⟶}\hat{Q}$, ${\hat{P}}_{n}\overset{s}{⟶}\hat{P}$, and ${\hat{R}}_{n}\overset{s}{⟶}\hat{R}$ then $\hat{Q}\hat{P}=\hat{R}$.

For weak convergence, however, we need to use every pair of vectors |u⟩,|v⟩∈𝐕 and form the inner product $q_{n}(u,v)\equiv \left\langle u|{\hat{Q}}_{n}|v\right\rangle$. In this case if $q_{n}(u,v)\to q(u,v)$ as n→∞ for all |u⟩,|v⟩∈𝐕, then ${\hat{Q}}_{n}$ converges *weakly* on 𝐕 to $\hat{Q}$ where $\hat{Q}|v\rangle =|q(v)\rangle$, and we write ${\hat{Q}}_{n}\overset{w}{⟶}\hat{Q}$ on 𝐕. Again, if a sequence converges strongly, then it also converges weakly to the same limit point. Also note that it is not valid to use a weak limit point $\hat{Q}$ in the same way as the terms of the sequence and, in particular, weak limits do not necessarily preserve the algebra of the sequences, i.e. if ${\hat{Q}}_{n}{\hat{P}}_{n}={\hat{R}}_{n}$, ${\hat{Q}}_{n}\overset{w}{⟶}\hat{Q}$, ${\hat{P}}_{n}\overset{w}{⟶}\hat{P}$, and ${\hat{R}}_{n}\overset{w}{⟶}\hat{R}$ then $\hat{Q}\hat{P}$ is not necessarily equal to $\hat{R}$.

### A.2.Rigged Hilbert space

We briefly summarize the salient points of the rigged Hilbert space framework. To avoid obscuring the broad structure with excessive details, we treat vectors |v⟩∈𝐕 in an abstract vector space 𝐕 interchangeably with their realization as functions in a corresponding linear space of functions via a mapping such as the inner product v(x)=⟨x|v⟩ in 𝐕 where x represents one or more independent variables. We also treat a linear functional f[g] as equivalent to an inner product via f[g]=⟨f|g⟩ or $f[g]=\int f^{*}(x)g(x)dx$.

A *distribution* Δ(x) is defined to give the limit point as n→∞ of a sequence of linear functionals $\Delta_{n}[\tau ]$ composed of representative functions $\Delta_{n}(x)$ over the elements τ(x) of a family of *test* functions. An important example is a test function that is differentiable to all orders on some domain (i.e. they are ‘smooth’ in this sense), and collectively they form Schwartz space ${𝚽}_{\tau }=\left\{\tau (x)\right\}$. The representative functions $\Delta_{n}(x)$ are elements of the space ${𝚽}_{\tau }^{\prime }=\left\{\Delta_{n}(x)\right\}$ which is the *dual* space of ${𝚽}_{\tau }$, where dual refers to the partner f(x) in the functional f[τ] for any $\tau (x)\in {𝚽}_{\tau }$. The dual space ${𝚽}_{\tau }^{\prime }$ is defined to be closed for all Cauchy sequences within it (i.e. it contains the limit points of all Cauchy sequences), and so the distributions are included in ${𝚽}_{\tau }^{\prime }$ accordingly.

The *rigging* of a Hilbert space H makes use of its *separability* property which hinges on the concept of dense subspaces. The space ${𝚿}^{c}$ formed by adding to an arbitrary vector space 𝚿 the limit points of all its Cauchy sequences is called the closure of 𝚿, 𝚿 is said to be a dense subspace of ${𝚿}^{c}$, and ${𝚿}^{c}$ is said to be separable in the sense that one can identity 𝚿 as a dense subset of it. The separability property of H [5] ensures that it contains ${𝚽}_{\tau }$ as a dense subset of test functions, which is automatically associated with a dual space ${𝚽}_{\tau }^{\prime }$ of distributions. This relationship is summarized by

$$\begin{array}{r} {𝚽}_{\tau }\subset H\subset {𝚽}_{\tau }^{\prime } \end{array}$$

and the structure it represents is called a rigged Hilbert space.
